# MiR-34a and miR-206 act as novel prognostic and therapy biomarkers in cervical cancer

**DOI:** 10.1186/s12935-017-0431-9

**Published:** 2017-06-09

**Authors:** Ai-Hua Chen, Yu-E Qin, Wen-Fan Tang, Jing Tao, Hua-mei Song, Manzhen Zuo

**Affiliations:** Department of Gynecology and Obstetrics, The People’s Hospital of China, China Three Gorges University, The First People’s Hospital of Yichang, Yichang, Hubei 443000 China

**Keywords:** MiR-34a, MiR-206, qRT-PCR, Cervical cancer

## Abstract

**Background:**

Recent evidence indicated that the aberrant expression of microRNA plays a crucial role in the development of cervical cancer. The overall shorter survival was strongly related to the abnormal expression of microRNA-34a (miR-34a) and microRNA-206 (miR-206), which target B cell lymphoma-2(Bcl2) and c-Met. Hepatocyte growth factor (HGF)/c-Met pathway is related to the occurrence, development and prognosis of cervical cancer, and c-Met is significantly overexpressed in cervical squamous cell carcinoma. Bcl2 is also considered to be a promising target for developing novel anticancer treatments.

**Methods:**

In this study, we detect the expression of miR-34a and miR-206 in the cervical cancer tissue through quantificational real-time polymerase chain reaction (qRT-PCR) assay, and the expression of Bcl2 and c-Met from cervical cancer tissue were detected by immunohistochemistry.

**Results:**

The expression of miR-34a and miR-206 were down-regulated in the cervical cancer tissue through qRT-PCR assay. As target genes of miR-34a and miR-206, Bcl2 and c-Met were up-regulated in cervical cancer tissues through qRT-PCR assay and immunohistochemistry. Kaplan–Meier and log-rank analysis revealed that down-regulated expression of miR-34a and miR-206 were strongly related to shorter overall survival. Multivariate Cox proportional hazards model for all variables that were statistically significant in the univariate analysis demonstrated that miR-34a (*P* = 0.038) and miR-206 (*P* = 0.008) might be independent prognostic factors for overall survival of patients suffering from cervical cancer.

**Conclusions:**

The up-regulation of Bcl2 and c-Met promotes the cervical cancer’s progress, and the expression of miR-34a and miR-206 significantly correlated with the progression and prognosis in cervical cancer. All of these suggested that miR-34a and miR-206 might be the novel prognostic and therapy tools in cervical cancer.

**Electronic supplementary material:**

The online version of this article (doi:10.1186/s12935-017-0431-9) contains supplementary material, which is available to authorized users.

## Background

Cervical cancer is one of the most commonly diagnosed tumors and the main leading cause of tumor death among women especially in developing countries. Lymph node metastasis and local or regional relapse are the primary causes of death in cervical cancer patients. As a protooncogene, c-Met encodes the growth factor receptor for HGF, demonstrates the activity of tyrosine kinase and induces the movement, proliferation and invasion of epithelial cells [1–3]. The activation of HGF/c-Met signal pathway plays an important role in the occurrence and development of human tumors [4–12]. Several studies have shown that c-Met was significantly overexpressed in cervical squamous cell carcinoma and the HGF/c-Met pathway was related to the occurrence, development and prognosis of cervical cancer [13–15]. Moreover, c-Met gene also was thought as a biomarker which was used to evaluate the biological behavior and clinical outcome of cervical cancer [16]. All of these suggest that interference with c-Met’s activation may provide an effective approach for cervical cancer’s treatment, and the expression level of c-Met is an important factor for the diagnosis and prognosis of cervical cancer.

Bcl2 was first discovered as an oncogene in B cell malignancies. Bcl2 is one of the most prominent anti-apoptotic proteins and contributes to the tumourigenesis and resistance to current anticancer drugs. Due to the central role in apoptosis regulation, Bcl2 is a promising target for developing the novel anticancer treatments. Recently, several studies have demonstrated that Bcl2-inhibitors may be very beneficial when combined with other targeted agents in solid tumor treatment [17–19].

As noncoding RNA, microRNAs are capable to bind the 3′-untranslated region (UTR) of specific genes and act as the inhibitor of corresponding mRNA targets translation. MiRNAs play crucial roles in various biological processes such as differentiation, proliferation and apoptosis [18–22]. Several studies have been addressing the impact of miRNAs in tumor development either by acting as oncogenes or tumor suppressor genes [23–25, 27–29]. And previous studies have demonstrated that both Bcl2 and c-Met are the targets of miR-34a and miR-206 [30–32, 35–40].

In this study, we found that the aberrant expressed microRNAs, miR-34a and miR-206, may play crucial roles through targeting Bcl2 and c-Met genes in cervical cancer tissue. It suggests that miR-34a and miR-206 are potential tools in the prognostic and therapy of cervical cancer.

## Methods

### Ethics statement

All patients agreed to participate in the study and gave written informed consent. This study was approved by the medical ethics committee of China Three Gorges University and complied with the Declaration of Helsinki.

### Patients and tissue specimens

A total of 41 cervical cancer tissues and matched adjacent normal tissues were obtained from patients who underwent surgery in the first hospital of Yichang city, China. The tissue samples were frozen and stored at −80 °C after surgical removal until use. The clinical histopathological diagnosis of tissue samples were approved by pathologists. The determination of the clinical stage was performed according to the International League of Gynecology and Obstetrics (FIGO).

### Extraction of RNA and qRT-PCR

In brief, the total RNA was extracted from collected samples with using TRIzol reagent (Invitrogen, Carlsbad, CA, USA). MiraMasTM Kit (Bioo Scientific, USA) was applied to perform the reverse-transcription reactions. RT-PCR analyses for genes were done with using SYBR green (Takara, Japan) on applied biosystems 7500 real-time PCR system, U6 gene was applied as references (Additional file 1). Moreover, the relative expressions of miRNAs were analyzed with the comparative cycle threshold (CT) method-fold change (2^−ΔΔCT^).

### Immunohistochemistry

All of the cervical tissue specimens were fixed with 10% neutral formalin embedded with paraffin, and serially sectioned at 5 μm. The sections were mounted onto the histostick-coated slides. Four or five adjacent ribbons were collected for histopathological analysis and for immunohistochemical staining. Histopathological diagnosis for tumor tissues was made according to cellular morphological changes and tissue architecture using the previously established criteria.

The biotin–streptavidin complex method was used for the immunostaining of c-Met, Bcl2. In brief, after dewaxing, inactivating the endogenous peroxidase activity and blocking cross-reactivity with normal non-immune goat serum, the sections were incubated at 4 °C overnight with a diluted solution of the first antibodies. The location of the primary antibodies was achieved by the subsequent application of a biotin-conjugated IgG (2^d^ antibody), a streptavidin-peroxidase. The color was visualized with DAB and the cellular nuclei were counterstained with instant hematoxylin. Negative controls were stablished by replacing the primary antibody with normal isotype serum.

### Follow-up

The patients were followed every 6 months for 2 years and then annually, thereafter. The total follow-up period was defined as the time from diagnosis to the date of death or the last censused date if the patients are still alive. There are all 41 patients that are included in the survival data analysis.

### Statistical analysis

All variables were analyzed by software SPSS version 17.0 (SPSS Inc, Illinois, USA). The Chi square test was used to investigate the association of miRNA expression with clinical parameters. The log-rank test and Kaplan–Meier method were applied for the evaluation of survival rate. Multivariate Cox regression was used to analyze the independent prognostic factors that were related to the survival of patients. Statistical significance was concluded at *P* < 0.05.

## Results

c-Met and Bcl2 up-regulated promote cervical cancer progress.

**Fig. 1:**
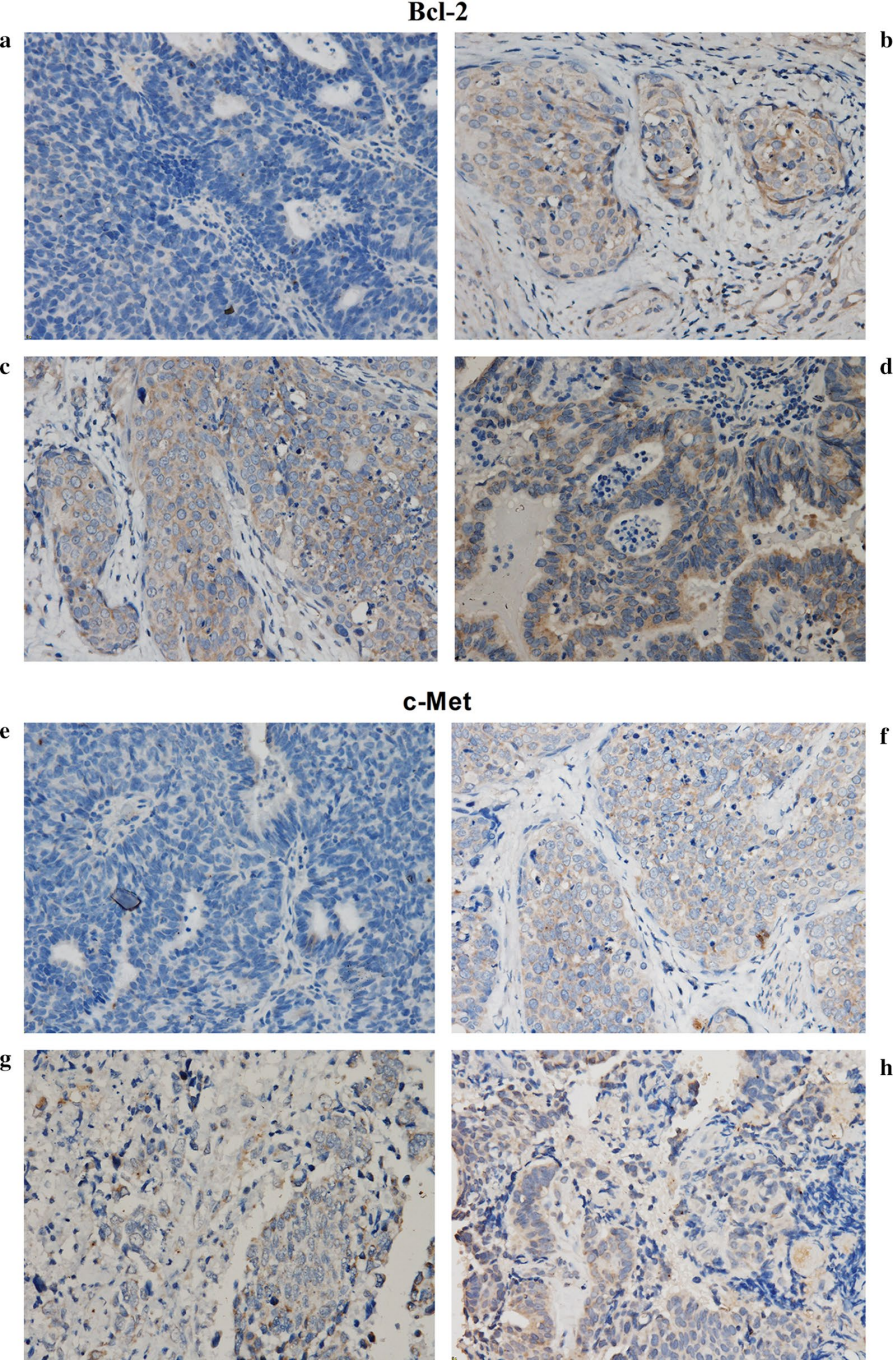
Comparison of Bcl2 and c-Met expression level between cervical cancer tissues and normal tissues. **a** and **e**: tumor-adjacent normal cervical tissue; **b** and **f**: Ib; **c** and **g**: IIIa; **d** and **h**: IIIb. **a**, **b**, **c**, **d**: Bcl2; **e**, **f**, **g**, **h**: c-Met

Consistent with previous researches [14, 15, 18, 19], c-Met and Bcl2 were up-regulated in cervical cancer tissues through immunohistochemistry in our study (Fig. 1). Patients with elevated expression of Bcl2 and c-Met tend to have lymph node metastasis (*P* = 0.000, *P* = 0.001), advanced stage (*P* = 0.00) (Table 1). These results indicated that Bcl2 and c-Met involved in the metastasis and progression in patients with cervical cancer. Inconsistent with other studies, elevated expression of Bcl2 and c-Met showed no significant correlation with advanced histological grade (*P* = 0.056, *P* = 0.06) in our study (Table 1). This may be due to the cases in our study are more moderate differentiated so that the correlation between the up-regulated Bcl2 and c-Met and the advanced histological grade is not obvious.

**Table 1:** Association of Bcl2 and c-Met expression with clinicopathological features

Variables	No. of cases	No. of expression of Bc12		No. of expression of c-Met		Bc12 (*P*)	c-Met (*P*)
		Low = 30	High = 11	Low = 29	High = 12		
Tumor size (cm)							
>4	18	17	1	17	1	0.011	0.005
≤4	23	13	10	12	11		
Histological grades							
Well differentiated	4	4	0	4	0	0.056	0.06
Moderate differentiated	34	25	9	24	10		
Poorly differentiated	3	1	2	1	2		
FIGO stage							
Ib–IIa	25	24	1	25	0	0.000	0.000
IIb–IIIa	12	6	6	4	8		
IIIb above	4	0	4	0	4		
Lymph node metasis							
Yes	17	7	10	7	10	0.000	0.001
No	24	23	1	22	2		

**Fig. 2:**
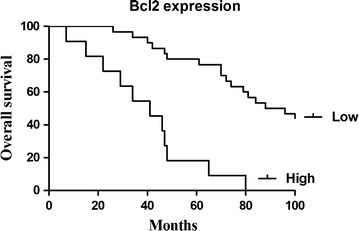
Survival analysis of cervical patients by Kaplan–Meier method (Bcl2)

**Fig. 3:**
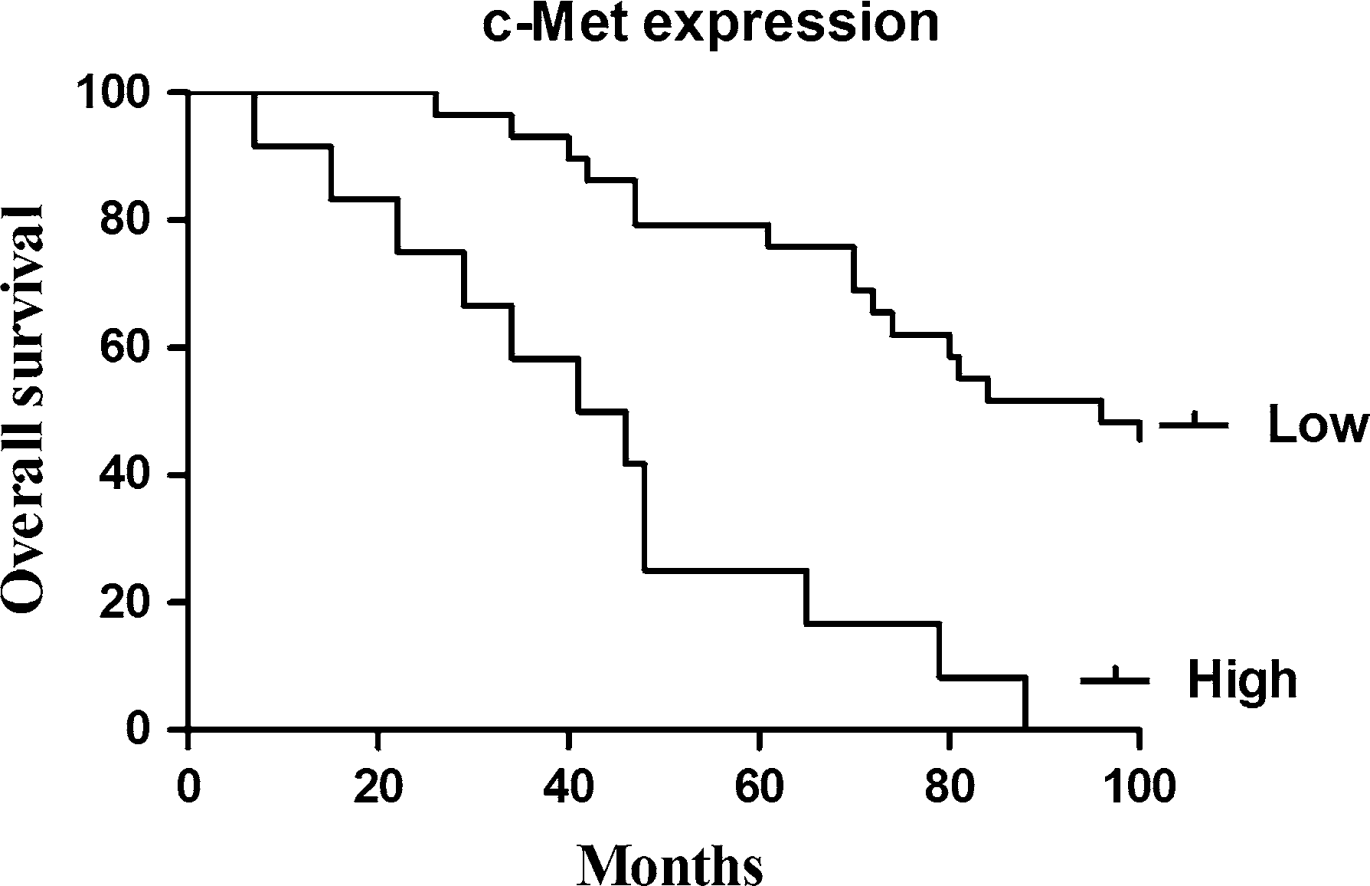
Survival analysis of cervical patients by Kaplan–Meier method (c-Met)

Kaplan–Meier and log-rank analysis revealed that the up-regulated expression of Bcl2 and c-Met were strongly related to shorter survival (Figs. 2, 3). Multivariate Cox proportional hazards model for all variables that were statistically significant in the univariate analysis demonstrated that high expression of Bcl2 and c-Met might be independent prognostic factors for the overall survival of the patients suffering from cervical cancer (Tables 2, 3).

**Table 2:** Univariate and multivariate analysis of prognostic parameters by Cox (Bcl2)

Clinicopathological characteristics	Relative risk (RR)	Univariate log-rank test (*P*)	Cox multivariable analysis (*P*)
Tumor diameter (cm)	1.220	0.03	0.791
Histological grades	0.422	0.04	0.188
FIGO stage	1.486	0.002	0.04
Lymph node metastasis	6.130	0.001	0.001
Bcl_2_ expression (high/low)	1.554	0.002	0.008

**Table 3:** Univariate and multivariate analysis of prognostic parameters by Cox (c-Met)

Clinicopathological characteristics	Relative risk (RR)	Univariate log-rank test (*P*)	Cox multivariable analysis (*P*)
Tumor diameter (cm)	1.148	0.042	0.801
Histological grades	0.409	0.056	0.202
FIGO stage	1.496	0.003	0.04
Lymph node metastasis	6.695	0.001	0.000
c-Met expression (high/low)	1.651	0.007	0.039

miR-34a and miR-206 expression significantly correlated with progression and prognosis in cervical cancer.

**Fig. 4:**
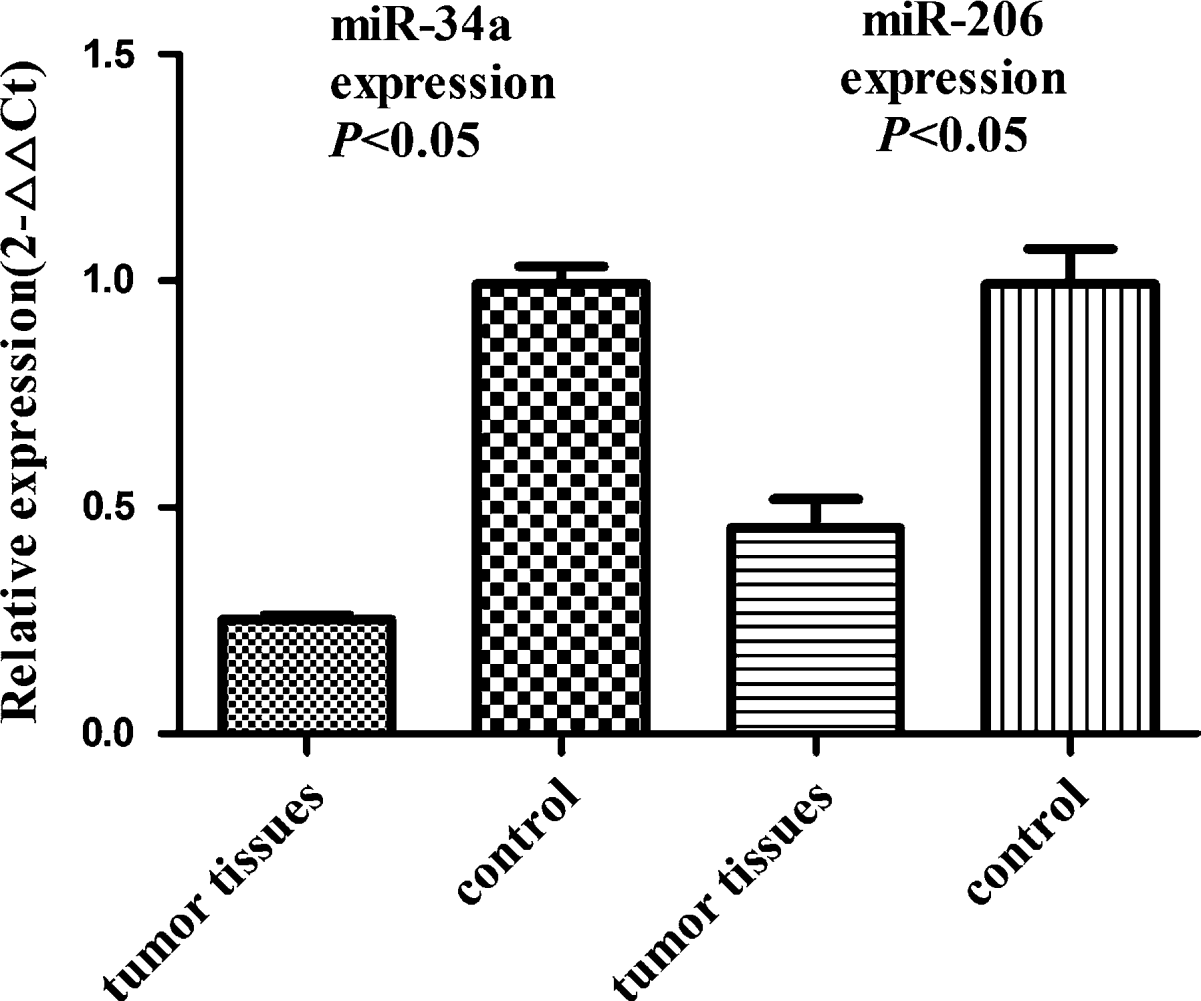
Comparison of miR-34a and miR-206 expression level between cervical cancer tissues and normal tissues

According to bioinformatics and the previous experiment results, both Bcl2 and c-Met are the targets of miR-34a and miR-206. Through Q-PCR, we found miR-34a and miR-206 were down regulated in cervical cancer tissue compare with tumor-adjacent normal cervical tissue (Fig. 4). Patients with decreased expression of miR-34a and miR-206 tend to have lymph node metastasis (*P* = 0.000, *P* = 0.001), advanced stage (*P* = 0.000, *P* = 0.000), advanced histological grade (*P* = 0.029, *P* = 0.023) (Table 4). These results indicate that miR-34a and miR-206 might be involved in the metastasis and progression in patients with cervical cancer.

**Table 4:** Association of miR-34a and miR-206 expression with clinicopathological features

Variables	No. of cases	No. of expression of miR-34a		No. of expression of miR-206		miR-34a (*P*)	miR-206 (*P*)
		Low = 21	High = 20	Low = 23	High = 18		
Tumor size (cm)							
>4	18	4	14	5	13	0.002	0.002
≤4	23	17	6	18	5		
Histological grades							
Well differentiated	4	0	4	0	4	0.029	0.023
Moderate differentiated	34	18	16	20	14		
Poorly differentiated	3	3	0	3	0		
FIGO stage							
Ib–IIa	25	24	1	7	18	0.000	0.000
IIb–IIIa	12	6	6	12	0		
IIIb above	4	0	4	4	0		
Lymph node metasis							
Yes	17	15	2	17	0	0.000	0.001
No	24	6	18	6	18		

**Fig. 5:**
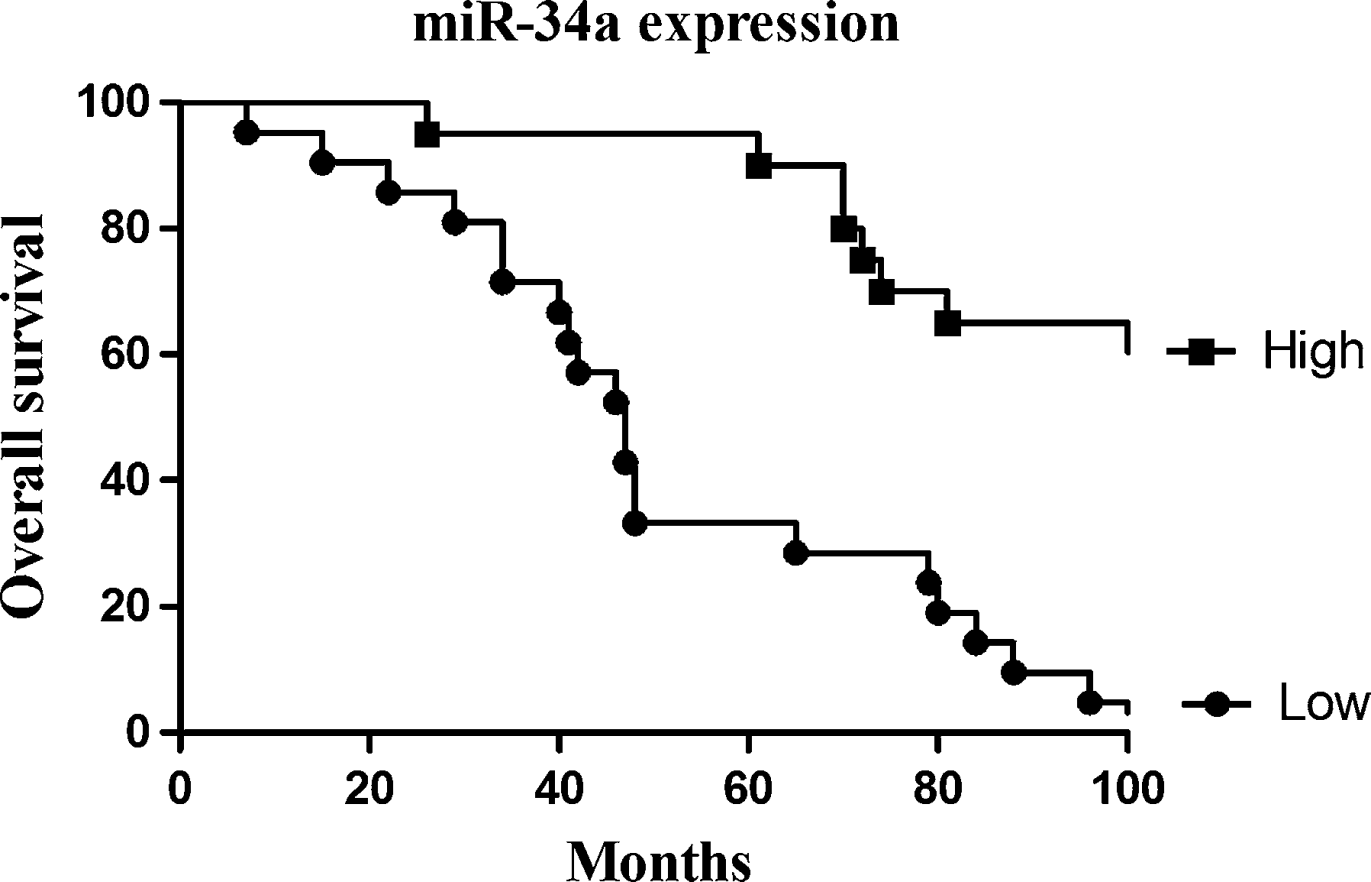
Survival analysis of cervical patients by Kaplan–Meier method (miR-34a)

**Fig. 6:**
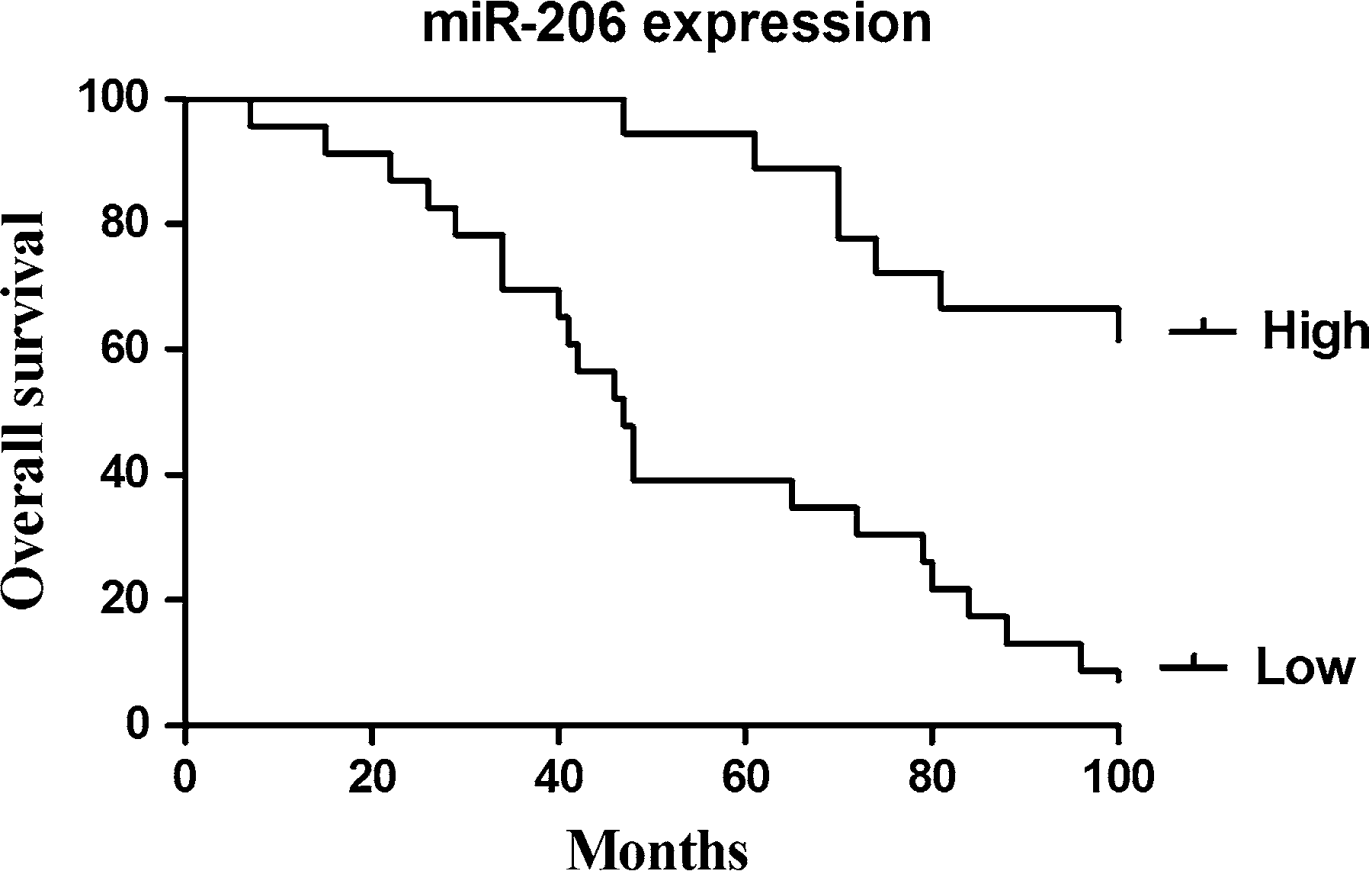
Survival analysis of cervical patients by Kaplan–Meier method (miR-206)

Kaplan–Meier and log-rank analysis revealed that the down-regulated expression of miR-34a and miR-206 were strongly related to a shorter overall survival (Figs. 5, 6). Multivariate Cox proportional hazards model for all variables that were statistically significant in the univariate analysis demonstrated that miR-34a (*P* = 0.038) and miR-206 (*P* = 0.008) might be independent prognostic factors for the overall survival of the patients suffering from cervical cancer (Tables 5, 6).

**Table 5:** Univariate and multivariate analysis of prognostic parameters by Cox (miR-34a)

Clinicopathological characteristics	Relative risk (RR)	Univariate log-rank test (*P*)	Cox multivariable analysis (*P*)
Tumor diameter (cm)	1.001	0.047	0.998
Histological grades	0.450	0.032	0.218
FIGO stage	1.757	0.002	0.038
Lymph node metastasis	6.567	0.001	0.001
miR-34a expression (high/low)	1.409	0.002	0.038

**Table 6:** Univariate and multivariate analysis of prognostic parameters by Cox (miR-206)

Clinicopathological characteristics	Relative risk (RR)	Univariate log-rank test (*P*)	Cox multivariable analysis (*P*)
Tumor diameter (cm)	1.170	0.036	0.775
Histological grades	0.478	0.041	0.238
FIGO stage	1.984	0.002	0.01
Lymph node metastasis	7.753	0.001	0.002
miR-206 expression (high/low)	1.885	0.002	0.008

## Discussion

Since bioinformatic studies have indicated that 30–50% of human genes’ expressions are probably controlled by miRNAs, it is conceivable that more miRNAs will play a critical role in cervical cancer’s occurrence and can potentially serve as biomarkers and targets for anticancer therapy [24, 25]. MiR-34a has been reported to be a key regulator of tumor suppression and down-regulated in several cancers, it involved in the occurrence and development of cancer [26]. MiR-34a is implicated in p53 network as the direct activator of p53. Wild type p53 induces the transcription of miR-34a which targets several molecules that are involved in cellular transformation and carcinogenesis [27–29]. The abnormal miR-34a expression has been revealed in breast cancer [31], colon cancer [32], cervical cancer [33], prostate cancer [34], esophageal squamous cell carcinoma [37], and lung cancer [38]. MiR-34a may act on its target genes to regulate the proliferation, apoptosis, invasion, metastasis and epithelial mesenchymal transition of cancer cells, exert inhibitory effects on the growth and metastasis of cancers [39]. In our research, we found miR-34a down-regulated in cervical cancer tissue and it might be involved in the metastasis and progression in patients with cervical cancer through targeting Bcl2 and c-Met which have been confirmed as targets of miR-34a [40, 41]. Besides Bcl2 and c-Met, other miR-34a target genes have been reported in different biological context, such as SIRT1, SIRT6, PNUTS, TGIF2 and HDAC1 which are closely related to cell proliferation, differentiation, apoptosis and other biological processes [42, 43]. As same as Bcl2 and c-Met, these genes also play crucial roles in the development of cancer. MiR-34a also has been found down-regulated in high-risk HPV infected tissues, which resulted from HPV-E6 expression [30]. HPV-E6 down-regulate the expression of miR-34a by degrading p53 through ubiquitin–proteasome system. High-risk HPVs are recognized to be the main cause for the development of cervical cancer. So the miR-34a down-regulation in cervical cancer tissue might be closely related with the high-risk HPVs infection. All of these suggested that miR-34a is a key tumor suppressor and a potential prognostic and therapy biomarker in cervical cancer.

As a skeletal muscle-enriched miRNA, miR-206 inhibits the proliferation of progenitor cells and promotes the myogenesis [44]. However, further studies showed that miR-206 is down-regulated in breast cancer, melanoma tumors and other various types of human cancers [45]. These researchers proposed that the down expression of miR-206 may be linked with cancer’s development. Several studies indicated that miR-206 induces G1 arrest in melanoma cell lines and function as a pro-apoptotic factor in HeLa cells through targeting Notch3 signal pathway [45–47]. MiR-206 also promoted the myogenic differentiation and blocked the tumor growth in xenografted mice by the down-regulation of Met tyrosine-kinase receptor, the product of the MET proto-oncogene [48]. Researchers further showed that the decrease in miR-206 expression is associated with an increase in oncogene CCND1, CCND2 and MMP-9, and also a decrease in p57. CCND1 and CCND2 are well-established oncogenes in many different cancers [49, 50]. MicroR-206 also acts as a tumor suppressor in bladder cancer and colorectal cancer via targeting YRDC and FMNL2, which are closely related to the tumor cell proliferation and EMT [51, 52]. Sun et al. found that the down-regulation of c-Met and Bcl2 by microRNA-206, can activates apoptosis, inhibits tumor cell proliferation, migration and colony formation in human lung cancer [47]. In our study, we also found that miR-206 down-regulated in cervical cancer tissues from patients, and the expression of c-Met and Bcl2 were up-regulated. And patients with decreased expression of miR-206 tended to have lymph node metastasis (*P* = 0.001), advanced stage (*P* = 0.000), advanced histological grade (*P* = 0.023).

Moreover, Kaplan–Meier and log-rank analysis revealed that the down-regulated expression of miR-34a and miR-206 were strongly related to shorter overall survival. Multivariate Cox proportional hazards model for all variables that were statistically significant in the univariate analysis demonstrated that miR-34a (*P* = 0.038) and miR-206 (*P* = 0.008) might be independent prognostic factors for the overall survival of patients suffering from cervical cancer. All of these results in our study suggested that miR-34a and miR-206 might be involved in the progression and prognosis of cervical cancer through targeting Bcl2 and c-Met. Besides Bcl2 and c-Met, which were significantly up-regulated in cervical cancer in our study, other target genes of miR-34 and miR-206 may also play crucial roles in the progression of cervical cancer tissue and which is the dominant role remain to be definite. Anyway, the down-regulation of miR-34a and miR-206 expression levels has the potential in acting as biomarker of the aggressive progression and the poor prognosis in cervical cancer.

## Conclusion

Based on previous studies which have demonstrated that Bcl2, c-Met are closely related with cervical cancer progression, we found their regulators, miR-34a and miR-206, also play important roles in the development and prognosis of cervical cancer. Patients with decreased expression of miR-34a and miR-206 tended to have lymph node metastasis, advanced stage, advanced histological grade and shorter survival in our study. It suggested that miR-34a and miR-206 also have the potential in acting as biomarkers of aggressive progression and poor prognosis in cervical cancer.

### Additional file

**Additional file 1.** Sequence of the primers used in this study.

## Abbreviations

miRNA: microRNA
miR-34a: microRNA-34a
miR-206: microRNA-206a
Bcl2: B-cell lymphoma-2
HGF: hepatocyte growth factor
qRT-PCR: quantificational real-time polymerase chain reaction
