# Supplementary figures and images for: MiR-34a and miR-206 act as novel prognostic and therapy biomarkers in cervical cancer
Source: Cancer Cell Int. 2017 Jun 9;17:63. doi: 10.1186/s12935-017-0431-9 (PMC5466768; doi:10.1186/s12935-017-0431-9)

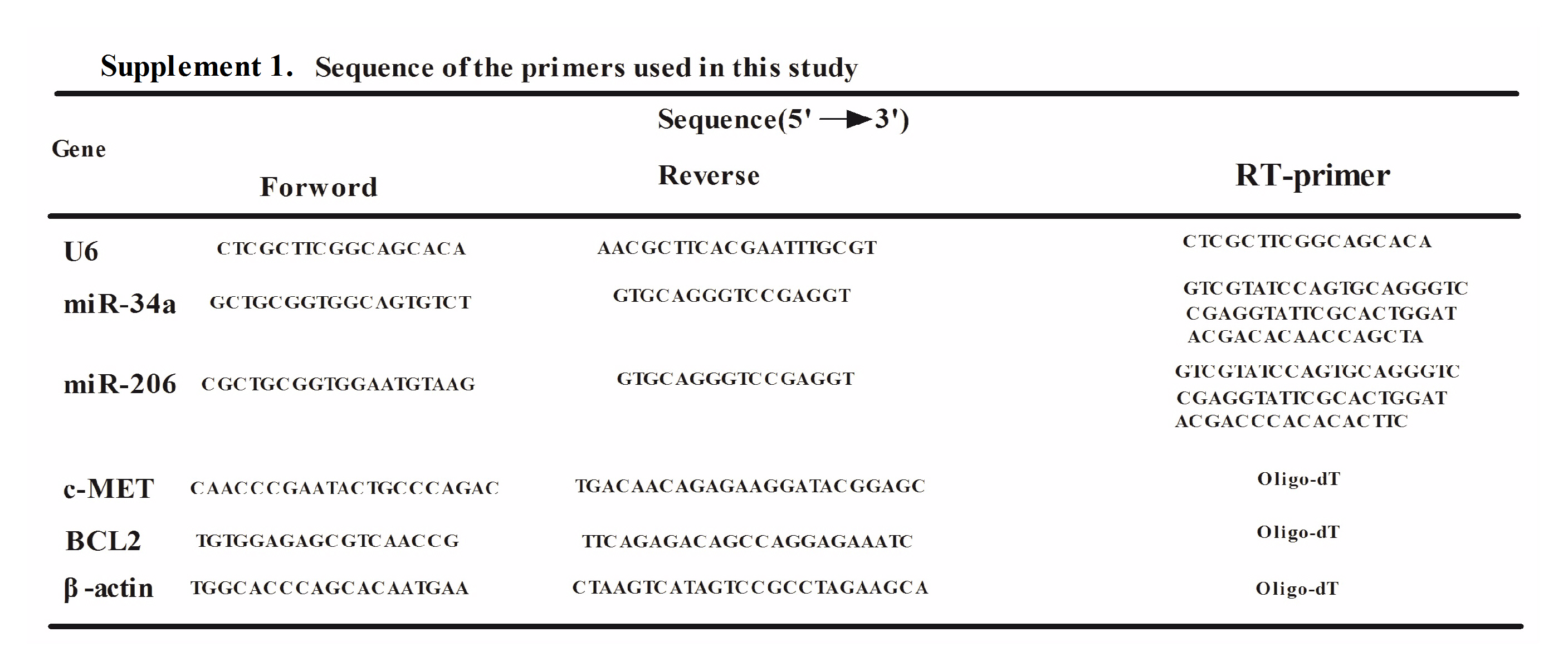

Supplement: Supplementary file 1 — Additional file 1. Sequence of the primers used in this study. [file 12935_2017_431_MOESM1_ESM.jpg]
